# Draft genome sequence of *Halobacillus campisalis* strain ASL-17

**DOI:** 10.1128/mra.00692-23

**Published:** 2024-01-11

**Authors:** Anushree Srivastava, Michael Christopher Macey, Terry J. McGenity, Karen Olsson-Francis

**Affiliations:** 1 AstrobiologyOU, The Open University, Milton Keynes, United Kingdom; 2 School of Life Sciences, University of Essex, Colchester, United Kingdom; Montana State University, Bozeman, Montana, USA

**Keywords:** bacteria, halophiles, genomics

## Abstract

We report here the genome sequence of moderately halophilic *Halobacillus campisalis* ASL-17, isolated from hypersaline sediment from the Yellow Sea, Korea. The bacterium was Gram variable, oval or coccoid, and mesophilic. The genome of *H. campisalis* ASL-17 has 3.8 Mbp, with 3,910 coding sequences, 76 RNAs, and 41.3% G + C content.

## ANNOUNCEMENT

Spring et al. (1) first described the genus *Halobacillus,* which belongs to the family *Bacillaceae* (2, 3) within the phylum *Firmicutes* (synonym *Bacillota*, 3). Genus *Halobacillus* comprises 27 species: *Halobacillus halophilus, Halobacillus litoralis*, *Halobacillus trueperi* (1), *Halobacillus thailandensis* (4), *Halobacillus salinus* (5), *Halobacillus karajensis* (6) *Halobacillus locisalis* (7), *Halobacillus styriensis* (8), *Halobacillus aidingensis* and *Halobacillus dabanensis* (9), *Halobacillus yeomjeoni* (10), *Halobacillus profundi* and *Halobacillus kuroshimensis* (11), *Halobacillus campisalis* (12), *Halobacillus faecis* (13), *Halobacillus mangrovi* (14), *Halobacillus alkaliphilus* (15), *Halobacillus seohaensis* (16), *Halobacillus naozhouensis* and *Halobacillus salsuginis* (17), *Halobacillus hunanensis* (18), *Halobacillus sediminis* (19), *Halobacillus andaensis* (20), *Halobacillus salicampi* (21), *Halobacillus massiliensis* (22), *Halobacillus marinus* (23), *Halobacillus ihumii* (24), and *Halobacillus fulvus* (25). Members of the genus *Halobacillus* are moderate to highly halophilic (13, 26), which makes them ideal candidates for biotechnological (27) as well as for astrobiological investigations (28). Many halophiles produce pigments (or carotenoids) to prevent photooxidative damage (29), endospores to survive a wide variety of physicochemical stresses [e.g., (30)], and synthesize osmoprotective compounds (31).

*H. campisalis* strain ASL-17 was isolated from hypersaline sediment from the Yellow Sea, Korea, by Yoon et al. (12). The polyphasic characterization revealed that *H. campisalis* strain ASL-17 is Gram-positive or variable, cocci or oval-shaped, and light-yellow in color. It grew optimally in the presence of approximately 8% (wt/vol) NaCl, at pH 7.0–8.0 and 37°C (12). The most distinguishing feature of the strain ASL-17 is that the cell wall peptidoglycan is composed of meso-diaminopimelic acid, unlike other *Halobacillus* (12).

For genome sequencing, freeze-dried cells of *H. campisalis* strain ASL-17 (strain CCUG 54360) were purchased from the Culture Collection University of Gothenburg Sweden (https://www.ccug.se). To culture, freeze-dried cells were first rehydrated using 0.5 mL of marine broth liquid medium marine broth (or DIFCO 2216), as recommended by the Leibniz Institute DSMZ-German Collection of Microorganisms and Cell Cultures (https://www.dsmz.de/) for the type species *H. halophilus* and mixed well. Then aliquots of the broth were transferred onto agar plates. The plates were then incubated at 30°C until growth appeared. A pure culture was obtained *via* repeated streaking, confirmed based on the uniformity of colony morphology. For genome sequencing, cultures were removed from the plate and resuspended into the barcoded bead tube provided by MicrobesNG (https://microbesng.com/) using the sterile loop. The tube was inverted 10 times, sealed, and returned to MicrobesNG at room temperature, with a guarantee that the delivery would arrive within 2 days of making the stock. Genomic DNA extraction and whole-genome sequencing were carried out by MicrobesNG. Genomic DNA libraries were prepared by MicrobesNG using the Nextera XT Library Prep Kit (Illumina, San Diego, USA) following the manufacturer’s protocol with the following modifications: input DNA was increased twofold, and PCR elongation time was increased to 45 s. DNA quantification and library preparations were carried out on a Hamilton Microlab STAR automated liquid handling system (Hamilton Bonaduz AG, Switzerland). Pooled libraries were quantified using the Kapa Biosystems Library Quantification Kit for Illumina. The genome libraries were sequenced by Illumina HiSeq technology and a 250 bp paired-end protocol. The assembly metrics were calculated using QUAST (Quality Assessment Tool for Genome Assemblies). Trimmed reads were produced using Trimmomatic (v0.30) with a sliding window quality cutoff of Q15. *De-novo* assembly was performed using SPAdes (v3.7; default settings) (32, 33). Coverage of 53-fold was achieved, calculated using BWA, SAMtools (v0.1.19), and BEDTools genomecov (v2.2.7) with default settings (34–36). Functional annotation of genes was performed using the RAST server (37) with the SEED database (38).

The assembled genome sequence of *H. campisalis* ASL-17 yielded 3,828,543 bp distributed in 43 contigs (scaffold N50 size = 4,52,793 bp). The G + C content was 41.3%, with 448 subsystems, 3,910 CDS, 67 tRNAs, and 1 tmRNA. The most represented RAST subsystem features, and the respective gene clusters (>100 gene clusters) were carbohydrates (430), amino acids and derivatives (429), protein metabolism (234), cofactors, vitamins, prosthetic groups, pigments (214), fatty acids, lipids, and isoprenoids (158), RNA metabolism (143), cell wall and capsule (128), stress response (116), nucleosides and nucleotides (112), and DNA metabolism (104). Notably, the genome of strain ASL-17 possesses a number of anaerobic respiratory reductases that potentially act as terminal electron acceptors in anaerobic respiration, including thiosulfate reductase, anaerobic dimethyl sulfoxide reductase, anaerobic sulfite reductase, dissimilatory sulfite reductase, and ferric and arsenate reductases.

## Data Availability

The draft genome sequence of Halobacillus campisalis type strain ASL-17 was deposited to GenBank under WGS accession JAPVRC000000000.
